# Comparative efficacy of different modalities of transcranial magnetic stimulation for treating Parkinson’s disease with depression: a systematic review and network meta-analysis

**DOI:** 10.3389/fneur.2025.1627932

**Published:** 2025-07-31

**Authors:** Zhichen Wang, Senlin Chen, Qianhong Zhu, Shun Chen, Yulong Zou, Gengzhao Chen, Qiuyi Luo, Sai’e Huang

**Affiliations:** ^1^Rehabilitation Hospital Affiliated to Fujian University of Traditional Chinese Medicine, Fuzhou, China; ^2^Fujian University of Traditional Chinese Medicine, Fuzhou, China; ^3^Key Laboratory of Cognitive Rehabilitation of Fujian Province, Fuzhou, China; ^4^Key Laboratory of Rehabilitation Technology of Fujian Province, Fuzhou, China

**Keywords:** depression, network meta-analysis, Parkinson’s disease, parameters, transcranial magnetic stimulation

## Abstract

**Objective:**

Parkinson’s disease (PD) is a prevalent neurodegenerative disorder. However, systematic comparisons of various transcranial magnetic stimulation (TMS) modalities for treating depression in patients with PD remain limited. This study aimed to evaluate the therapeutic effects of different TMS modalities on depression in patients with PD.

**Methods:**

A systematic search following PRISMA guidelines was conducted in the following databases: Cochrane Library, Embase, PubMed, Web of Science, CNKI, WanFang, VIP, and CBM, covering literature up to April 1, 2025. Included studies were randomized controlled trials (RCTs) evaluating TMS interventions in PD patients with depression. Risk of bias was assessed using the Cochrane Risk of Bias 2.0 (ROB 2.0) tool. Comparative effectiveness analysis was conducted using STATA 17.0.

**Results:**

A total of thirty-five RCTs involving 2,353 participants were included, evaluating high-frequency repetitive transcranial magnetic stimulation (HF-rTMS) and low-frequency repetitive transcranial magnetic stimulation (LF-rTMS) compared to sham stimulation and conventional rehabilitation therapy (CRT). The results showed that LF-rTMS [standardized mean difference (SMD) = −2.46, 95% confidence interval (−3.62, −1.29)], HF-rTMS [SMD = −2.05, 95% CI (−3.16, −0.94)] significantly improved depressive symptoms.

**Conclusion:**

The network meta-analysis indicates that both HF-rTMS and LF-rTMS may be considered as effective adjunctive therapy to improve depression in patients with PD, with LF-rTMS potentially showing superior efficacy in improving depressive symptoms. Parameters, such as total pulses no more than 1,200, may optimize outcomes. However, further high-quality RCTs are required to validate these findings and refine optimal treatment protocols.

**Systematic review registration:**

https://www.crd.york.ac.uk/prospero/, identifier CRD42024564867.

## Introduction

Parkinson’s disease (PD) is a neurodegenerative disorder prevalent among middle-aged and elderly populations (1). The primary pathological characteristic of PD is the degeneration and loss of dopaminergic neurons in the substantia nigra-striatal pathway (2), resulting in motor symptoms (MS) and non-motor symptoms (NMS). Resting tremor, bradykinesia, and rigidity are predominantly features of MS, while NMS typically present as depression, hyposmia, cognitive impairment, sleep disorders, and constipation (3). Furthermore, the appearance of non-motor symptoms often precedes motor symptoms and dominates as PD progresses, exacerbating the burden on PD patients and complicating their management strategies (4). According to the Global Burden of Disease (GBD) report, approximately 6.1 million individuals were diagnosed with PD worldwide, and an additional 1.02 million new cases were reported in 2017, with this number continuing to rise alongside global aging trends (5–7). Moreover, it is foreseeable that by 2030, the number of PD patients is estimated to reach 4.94 million in China, with the burden caused by PD expected to rise substantially over the coming decades (8, 9).

It is widely recognized that depression represents the most prevalent mood disorder in PD patients, with at least one-third of individuals experiencing symptoms of depression (10, 11). This condition significantly impairs patients’ quality of life and may lead to varying degrees of disability (12–14). Consequently, early detection and timely intervention of depression are crucial to ensure positive treatment effects. Depression may be associated with the decreased dopamine levels in the anterior cingulate cortex (15). Pharmacological treatments, including dopamine agonists, selective serotonin reuptake inhibitors (SSRIs), and serotonin/norepinephrine reuptake inhibitors (SNRIs), have been employed to alleviate PD with depression (PD-D) patients (16, 17). However, the tolerance and adverse effects of drug therapy undermine the efficacy of these treatments (18).

Clinically, transcranial magnetic stimulation (TMS) modalities are typically classified according to stimulation paradigms and neurophysiological effects, including single-pulse TMS, paired-pulse TMS, deep TMS, and repetitive TMS (rTMS). Among these, rTMS induces small intracranial electric currents by stimulating neurons through alternating magnetic fields, which has been widely recognized for its safety and efficacy. It influences neurotransmitter activity and synaptic plasticity, for instance, it enhances the secretion and release of endogenous dopamine in the ipsilateral striatum, resulting in sustained alterations of neural circuits and subsequent improvement in depressive symptoms. Thus, it serves as a valuable adjunctive therapy within clinical treatment regimens for depression (19–21). The pattern of rTMS varies according to specific frequencies and patterns. Low-frequency stimulation (below 1 Hz) suppresses neuronal activity and reduces cortical excitability. In contrast, high-frequency stimulation (above 5 Hz) promotes neuronal depolarization, thereby increasing cortical excitability (22–24). Intermittent theta burst stimulation delivers high-frequency pulses within a short time frame, mimicking theta rhythms to achieve excitatory effects. Bilateral rTMS combines high-frequency stimulation targeting the left dorsolateral prefrontal cortex (LDLPFC), with low-frequency stimulation targeting the right dorsolateral prefrontal cortex (RDLPFC), modulating both excitatory and inhibitory effects across hemispheres. Aftanas et al. (25) reported that high-frequency rTMS (HF-rTMS) to be significantly more effective than placebo in improving motor and mood symptoms in PD patients. Zhuang et al. (26) demonstrated that low-frequency rTMS (LF-rTMS) significantly improved cognitive function and alleviated depression in PD patients, resulting in stable and long-term therapeutic benefits. Chen (27) comparing the effects of HF-rTMS and LF-rTMS on PD-D patients, concluded that although both modalities effectively alleviate depression, LF-rTMS exhibits superior therapeutic efficacy.

However, rTMS setting parameters which include stimulation frequency, location, and duration vary widely in clinical practice, so optimizing rTMS treatment protocols is a pressing issue. This study incorporates randomized controlled trials (RCTs) of rTMS with different frequencies and stimulation sites for treating PD patients with depression, aiming to assess the therapeutic efficacy and acceptability of various rTMS modalities.

## Methods

This systematic review and network meta-analysis was conducted following the Preferred Reporting Items of the Guidelines for Systematic Reviews and Meta-Analysis (PRISMA) (28). The protocol was registered on the International Prospective Register of Systematic Reviews (PROSPERO), number: CRD42024564867.

### Search strategy

We conducted a comprehensive literature search from database inception to April 1, 2025, retrieving relevant articles from Cochrane Library, Embase, PubMed, Web of Science, China National Knowledge Infrastructure (CNKI), Wanfang Data (WanFang), VIP Database (VIP), and Chinese Biomedical Literature Database (CBM). We thoroughly examined references cited in the included RCTs. Full texts of all included articles were obtained, and authors were consulted for additional information as required. The search strategy, included in Supplementary material: Appendix 1, was carried out using Medical Subject Heading (MeSH) terms and keywords.

### Eligibility criteria

Eligible studies were randomized controlled trials (RCTs) published in peer-reviewed journals. Participants were adults with a confirmed PD diagnosis based on the UK Parkinson’s Disease Society Brain Bank Clinical Diagnostic Criteria or the Movement Disorder Society Clinical Diagnostic Criteria. PD-D was diagnosed using standardized criteria, specifically the Diagnostic and Statistical Manual of Mental Disorders, 4th text revision (DSM-4), ensuring all included studies recruited patients with a formal diagnosis of depression, supplemented by validated rating scales such as the Hamilton Depression Rating Scale (HAMD) or the Beck Depression Inventory (BDI). Interventions included HF-rTMS or LF-rTMS, with control groups receiving sham TMS or conventional rehabilitation therapy (CRT) which encompassing pharmacological treatment, psychotherapy, exercise therapy. The use of antidepressants was permitted during the TMS treatment period in the included studies. The primary outcome was the change in the HAMD scores, and the secondary outcome including Part I of the Unified Parkinson’s Disease Rating Scale (UPDRS-I) and BDI scores.

### Exclusion criteria

To ensure data reliability, studies were excluded based on the following criteria: non-RCT designs, failed to provide adequate details on the diagnostic criteria for PD or the methods used to assess depression, presence of significant comorbidities, incomplete data, systematic reviews, and conference abstracts.

### Study selection and data extraction

Two authors independently extracted data from included studies using a standardized extraction form designed in Microsoft Excel. Specific details included study characteristics (first author’s name, publication year, country), population characteristics (gender, age, duration of condition, sample size, Hoehn–Yahr stage), intervention methods (frequency, location, intensity total pulses and treatment cycle), control measures and outcome measures.

### Risk of bias assessment

The risk of bias in the included RCTs was independently evaluated by two authors (S.L. Chen and H.Q. Zhu) using version 2 of the Cochrane Risk of Bias tool (RoB 2.0). The assessment focused on domains primarily affected by potential sources of bias, including the randomization process, deviations from intended interventions, missing outcome data, outcome measurement, and selective reporting of outcomes. Each domain was rated as ‘low risk,’ ‘some concerns,’ or ‘high risk,’ and the overall risk of bias was determined based on the judgments across all domains. Any discrepancies arising during the analysis were resolved through mutual consultation and discussion.

### Data analysis

We used Review Manager software version 5.4 (RevMan 5.4) and STATA software version 17.0 (STATA 17.0) to conduct statistical analyses of the included RCTs. The mean difference (MD) and standardized mean difference (SMD) were used for data effect sizes for continuous variables, with 95% confidence interval (CI) for combined estimates. Heterogeneity was quantified by using *I*^2^ and *p* values, where a combination of *I*^2^ < 50% and *p* ≥ 0.1 was interpreted as low heterogeneity, necessitating the use of fixed-effects models for aggregated effect sizes. In cases of substantial heterogeneity, random-effects models were applied instead. Sensitivity analyses and bias assessments were performed using STATA 17.0 to ensure the stability and accuracy of our findings. Network relationships were mapped using STATA 17.0, using the HAMD scores as outcome measures to compare the various interventions. Additionally, the relative efficacy of these interventions was assessed by a network meta-analysis.

## Results

### Study retrieval results

**Figure 1:**
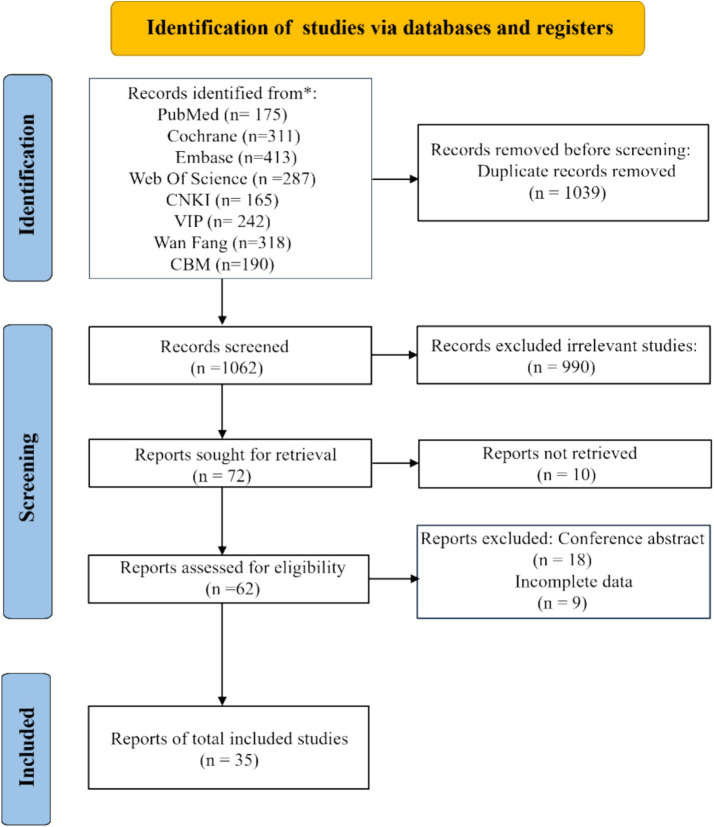
PRISMA flow diagram.

A total of 2,101 articles were initially retrieved from eight databases based on the search strategy. After thorough screening to exclude duplicates, irrelevant publications, studies on similar topics, conference papers, and incomplete data, 35 studies were eventually involved. The PRISMA flow diagram is illustrated in Figure 1.

### Study characteristics

The included studies were published between 2004 and 2025, primarily from Asia (*n* = 27), followed by Europe (*n* = 3), North America (*n* = 3), South America (*n* = 1), and Africa (*n* = 1). The studies included 2,353 participants (50.19% male, *n* = 1,181), with a mean age over 60 years, and most having a disease duration of 6 years or longer. No significant differences were found between groups in demographic and clinical characteristics. The 35 studies evaluated four types of interventions for depression, including HF-rTMS, LF-rTMS, sham rTMS, and CRT, with intervention durations ranging from 10 days to 8 weeks. Detailed patient data and rTMS parameters are presented in Tables 1, 2.

**Table 1:** Baseline demographic and clinical characteristics of patients enrolled in the included trials.

ID	Study (year)	Country	Treatment group				Control group			
			M/F	Ages	Duration of disease	H-Y	M/F	Ages	Duration of disease	H-Y
1	Aftanas et al. (25)	Russia	12/11	63.7 ± 8.8	7.0 ± 4.0	-	9/14	62.9 ± 7.1	5.6 ± 4.0	-
2	Alvaro (69)	USA	11/9	68.2 ± 8.0	-	-	11/4	66.2 ± 12.7	-	-
			9/5	62.7 ± 13.0	-	-				
			6/6	67.3 ± 12.7	-	-				
3	Boggio et al. (70)	Brazil	13	-	-	-	12	-	-	-
4	Brys et al. (71)	USA	9/5	59.6 ± 12.6	8.4 ± 5.2	-	11/4	64.0.0 ± 7.4	4.5 ± 2.2	-
			6/6	64.6 ± 12.3	7.7 ± 4.2	-				
			11/9	64.9 ± 68.0	7.3 ± 5.6	-				
5	Cai (72)	China	13/7	65.35 ± 5.92	7.40 ± 2.25	-	12/8	66.90 ± 4.85	7.75 ± 2.12	-
6	Chen et al. (73)	China	10/11	63.19 ± 8.16	6.60 ± 5.53	2.8 ± 0.79	9/9	66.61 ± 8.00	6.22 ± 3.96	3.16 ± 0.49
			10/11	65.81 ± 9.38	5.88 ± 5.29	2.98 ± 0.75				
7	Chen (27)	China	23/17	61.20 ± 4.36	5.66 ± 2.10	-	22/18	60.14 ± 5.72	6.09 ± 2.13	-
			21/19	62.34 ± 5.22	6.10 ± 1.47	-				
8	Chen et al. (74)	China	13/12	60.70 ± 8. 9	-	-	13/11	59.05 ± 6. 8	-	-
9	Cui and Zhang (75)	China	29/24	60.8 ± 5.2	3.2 ± 1.2	-	27/27	60.5 ± 4.5	3.0 ± 1.5	-
10	Feng et al. (76)	China	11/9	60.80 ± 6.8	-	-	10/10	58.01 ± 5.6	-	-
11	Fregni et al. (77)	USA	11/10	65.3 ± 7.8	-	-	15/6	66.0 ± 8.5	-	-
12	Guo (78)	China	18/20	65.91 ± 3.42	6.48 ± 2.08	-	19/19	66.57 ± 3.39	6.64 ± 2.21	-
			17/21	66.28 ± 3.55	6.15 ± 1.97	-				-
13	Guo et al. (79)	China	20/20	62.53 ± 4.17	2.76 ± 0.66	-	19/21	63.65 ± 5.62	2.80 ± 0.59	-
			22/18	63.20 ± 5.42	2.75 ± 0.61	-				
14	Han et al. (80)	China	15/20	64.8 ± 8.1	-	-	14/21	63.6 ± 8.1	-	-
15	Jiang et al. (81)	China	8/10	67.49 ± 7.83	-	-	11/7	65.49 ± 7.43	-	-
16	Jiang et al. (82)	China	14/14	62.7 ± 12.9	7 ± 5.33	-	12/17	64.3 ± 8.9	3 ± 3.70	-
17	Khedr et al. (83)	Egypt	7/9	61.82 ± 3.48	7.12 ± 3.48	-	6/2	60.21 ± 1.64	5.87 ± 4.08	-
18	Li (84)	China	15/15	65.3 ± 8.1	6.6 ± 5.3	-	16/14	66.5 ± 7.5	6.4 ± 4.9	-
			16/14	66.1 ± 7.6	6.1 ± 5.2	-				
19	Li et al. (85)	China	30/21	64.17 ± 5.42	6.17 ± 2.24	3.24 ± 0.87	31/20	64.02 ± 5.67	6.12 ± 2.13	3.19 ± 0.92
20	Liu et al. (86)	China	18/12	63.22 ± 10.94	6.17 ± 1.34	-	16/13	60.71 ± 12.29	5.65 ± 1.33	-
21	Makkos et al. (87)	Hungary	13/10	67 ± 9.63	6 ± 5.19	-	11/10	62 ± 5.93	5 ± 4.44	-
22	Pal et al. (88)	Hungary	6/6	68.5 ± 7.78	6 ± 4.81	-	5/5	67.5 ± 11.11	6.5 ± 5	-
23	Song et al. (89)	China	15/7	67.36 ± 6.99	6.18 ± 1.62	-	13/7	70.50 ± 6.76	6.77 ± 2.02	-
24	Tang et al. (90)	China	17/14	60.33 ± 7.67	5.49 ± 3.85	-	18/13	60.14 ± 7.63	5.66 ± 3.82	-
			15/15	60.18 ± 7.54	5.71 ± 3.88	-				
25	Wang et al. (91)	China	30/11	60.52 ± 2.35	-	-	32/9	60.15 ± 2.32	-	-
26	Wu (92)	China	4/6	63.90 ± 8.66	6.35 ± 3.64	2.5 ± 0.84	6/4	65.20 ± 4.24	5.60 ± 3.02	2 ± 0.74
27	Yang (93)	China	33/23	63.26 ± 7.39	6.77 ± 2.66	-	31/25	63.61 ± 7.85	6.97 ± 2.78	-
28	Yu et al. (94)	China	14/17	67.25 ± 6.71	2.76 ± 1.56	-	16/17	68.00 ± 7.56	2.64 ± 1.49	-
29	Zhang et al. (95)	China	14/16	62.36 ± 7.14	4.72 ± 2.91	-	8/7	65.85 ± 5.84	6.23 ± 4.02	-
30	Zhang (96)	China	22/18	60.0 ± 2.1	-	-	21/19	59.5 ± 2.3	-	-
31	Zhang (97)	China	6/5	66.0 ± 9.0	5.3 ± 3.2	2.4 ± 0.5	6/5	62.2 ± 7.3	5.8 ± 3.4	2.5 ± 0.4
			9/2	63.6 ± 9.1	4.6 ± 3.3	2.0 ± 0.7				
32	Zhang et al. (98)	China	24/14	65.38 ± 8.34	4.40 ± 1.45	2.36 ± 0.57	25/15	63.90 ± 7.75	4.25 ± 1.69	2.33 ± 0.57
33	Zhou et al. (99)	China	20/20	65.51 ± 6.14	-	-	22/18	65.14 ± 6.54	-	-
34	Zhu (100)	China	7/7	63.21 ± 7.29	6.67 ± 4.73	2.11 ± 0.63	7/7	61.57 ± 13.25	5.71 ± 3.77	2.18 ± 0.75
35	Zhuang et al. (26)	China	11/8	60.58 ± 9.21	5.86 ± 4.36	2 ± 0.74	7/7	61.57 ± 13.25	5.71 ± 3.77	2.25 ± 0.92

F, female; H-Y, Hoehn–Yahr stage; M, male.

**Table 2:** The parameter settings and characteristics of different rTMS modalities in the included trials.

ID	Intervention measure						Control measure	Outcomes
	Mode	Frequency	Location	Intensity	Total pulses	Treatment Cycle		
1	HF-rTMS	10 Hz	M1 + DLPFC	100-110%RMT	7,000	3w	sham rTMS	①②③
2	HF-rTMS	10 Hz	M1 + LDLPFC	80-90%RMT	3,000	2w	sham rTMS	②④
	HF-rTMS	10 Hz	M1 + sham DLPFC	90%RMT	1,000			
	HF-rTMS	10 Hz	sham M1 + LDLPFC	80-90%RMT	2,000			
3	HF-rTMS	15 Hz	LDLPFC	110%RMT	-	2w	sham rTMS	②⑤
4	HF-rTMS	10 Hz	DM1	-	2,000	10d	Sham rTMS	①
	HF-rTMS	10 Hz	LDLPFC	-	2,000			
	HF-rTMS	10 Hz	DM1 + LDLPFC	-	4,000			
5	HF-rTMS	5 Hz	LDLPFC	110%RMT	1,600	8w	CRT	①②③
6	HF-rTMS	5 Hz	M1 Hand	100%RMT	1,600	10d	sham rTMS	④
	LF-rTMS	1 Hz	M1 Hand	100%RMT	1,600			
7	HF-rTMS	5 Hz	RDLPFC	-	-	4w	sham rTMS	①
	LF-rTMS	0.5 Hz	LDLPFC	-	-			
8	HF-rTMS	5 Hz	LDLPFC	110%RMT	1,600	8w	CRT	①
9	LF-rTMS	0.5 Hz	LDLPFC	90-100%RMT	-	4w	CRT	①
10	LF-rTMS	1 Hz	RDLPFC	80%RMT	1,600	4w	sham rTMS	①
11	HF-rTMS	15 Hz	LDLPFC	110%RMT	-	2w	sham rTMS	②⑤
12	HF-rTMS	5 Hz	M1 Hand	100%RMT	1,600	10d	sham rTMS	①
	LF-rTMS	1 Hz	M1 Hand	100%RMT				
13	HF-rTMS	5 Hz	LDLPFC	90%RMT	-	2w	sham rTMS	①
	LF-rTMS	0.5 Hz	RDLPFC	90%RMT	-			
14	HF-rTMS	10 Hz	LDLPFC	120%RMT	2,000	15d	sham rTMS	④
15	LF-rTMS	1 Hz	-	-	180	15d	CRT	④
16	HF-rTMS	10 Hz	LDLPFC	100%RMT	1,200	10d	sham rTMS	①
17	HF-rTMS	20 Hz	bilateral parietal cortexes	80%RMT	2,000	2w	sham rTMS	②
18	HF-rTMS	5 Hz	RDLPFC	90-100%RMT	1,740	4w	sham rTMS	④
	LF-rTMS	0.5 Hz	LDLPFC	90-100%RMT	750			
19	HF-rTMS	5 Hz	LDLPFC	80%RMT	-	4w	sham rTMS	①
20	HF-rTMS	10 Hz	M1 + LDLPFC	90%RMT	3,000	10d	sham rTMS	①
21	HF-rTMS	5 Hz	M1	90%RMT	600	10d	sham rTMS	②③
22	HF-rTMS	5 Hz	LDLPFC	90%RMT	600	10d	sham rTMS	②③
23	HF-rTMS	10 Hz	DM1	90%RMT	1,000	10d	sham rTMS	④
24	HF-rTMS	5 Hz	RDLPFC	90-100%RMT	1,740	4w	sham rTMS	①
	LF-rTMS	0.5 Hz	LDLPFC	90-100%RMT	750			
25	HF-rTMS	25 Hz	LDLPFC	90%RMT	500	1 m	sham rTMS	①
26	LF-rTMS	1 Hz	RDLPFC	80%RMT	1,200	10d	sham rTMS	①③
27	HF-rTMS	25 Hz	RDLPFC	80%RMT	1,350	4w	sham rTMS	①
28	HF-rTMS	5 Hz	DDLPFC	80%RMT	1,600	1 m	sham rTMS	①
29	LF-rTMS	1 Hz	-	110%RMT	-	10d	sham rTMS	③
30	HF-rTMS	5 Hz	M1 Hand	110%RMT	1,600	-	CRT	①③
31	HF-rTMS	10 Hz	DM1	90%RMT	1,500	5d	sham rTMS	①
	HF-rTMS	5 Hz	LDLPFC	90%RMT	1,500			
32	HF-rTMS	5 Hz	LDLPFC	90%RMT	1,600	4w	sham rTMS	①
33	HF-rTMS	5 Hz	DDLPFC	-	-	10d	sham rTMS	①
34	LF-rTMS	1 Hz	RDLPFC	110%RMT	1,000	10d	sham rTMS	⑤
35	LF-rTMS	1 Hz	RDLPFC	110%RMT	1,200	10d	sham rTMS	⑤

AMT, active motor threshold; CRT, conventional rehabilitation therapy; DDLPFC, double dorsolateral prefrontal cortex; DLPFC, dorsolateral prefrontal cortex; HF-rTMS, high frequency repetitive transcranial magnetic stimulation; LF-rTMS, low frequency repetitive transcranial magnetic stimulation; LDLPFC, left dorsolateral prefrontal cortex; M1, primary motor cortex; RDLPFC, right dorsolateral prefrontal cortex; RMT, resting motor threshold; ①, Hamilton Depression Scale-17 (HAMD-17); ②, Beck Depression Inventory (BDI); ③, Part I of the Unified Parkinson’s Disease Rating Scale (UPDRS-I); ④, Hamilton Depression Scale-24 (HAMD-24); ⑤, Hamilton Rating Scale for Depression (HRSD).

### Risk of bias assessment

**Figure 2:**
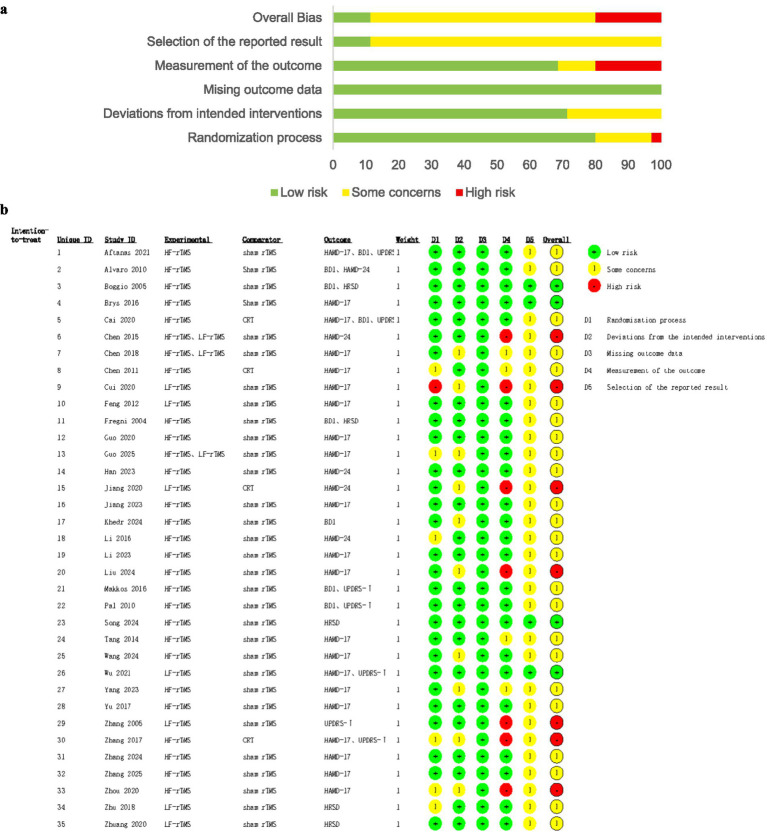
**(a)** Risk of bias assessment across domains. **(b)** Risk of bias assessment for individual studies.

This review is following the guidelines set out in The Cochrane Handbook of Systematic Reviews5.1.0. Figure 2a showed that 22 studies reported specific randomization methods, such as computerized randomization lists and random number tables, while 6 studies mentioned randomization without specifying the method used. Allocation concealment was described in 16 studies. Blinding was employed in 13 studies for both patients and trialists, and in 18 studies for outcome assessors. The outcome data for all studies were completed. A detailed summary of the risk of bias assessment using the ROB 2.0 tool for each included study is presented in Figure 2b. Among the 35 included studies, 4 studies were rated as ‘low risk’ overall, 24 studies as ‘some concerns’, and 7 studies as ‘high risk’. The most common sources of bias were in the domains of ‘selection of the reported result’, with 31 studies rated as ‘some concerns’ in these domains. Additionally, 6 studies were rated as ‘some concerns’ in the domain of ‘randomization process’, and the domain of ‘deviations from intended interventions’ had 10 studies rated as ‘some concerns’. In contrast, the domains ‘missing outcome data’ were generally at low risk. Studies with a high overall risk were mainly downgraded due to high risk in ‘measurement of the outcome’.

### Outcomes of the network meta-analysis

#### The network plots

To complete the comparison about the therapeutic efficacy of different rTMS modalities included in the review on the PD-D patients, we have constructed evidence network plots for the HAMD.

**Figure 3:**
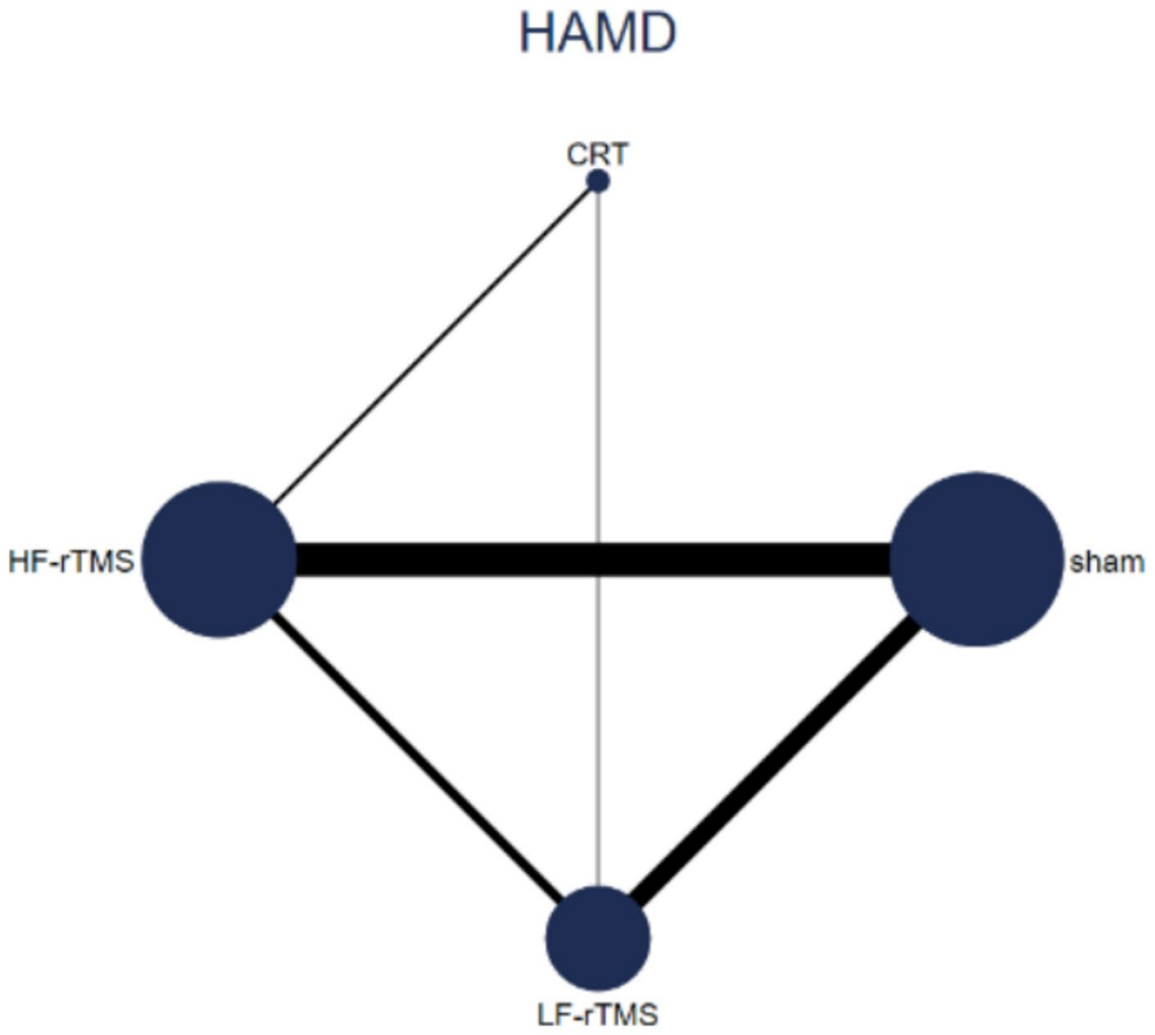
Evidence network diagram of efficacy comparison of different interventions for treating depression in patients with PD.

In this study, a total of 25 studies, involving 1,604 participants, related to HAMD were included, with 3 studies used CRT as the control group and remaining studies employed sham rTMS. In Figure 3, we can clearly observe the comparison between different modalities of rTMS and CRT. Each point in the figure represents an intervention method, and the number of cases involved in that particular intervention were presented by the size of the point proportional. The connecting lines indicate that the two intervention methods can be directly compared, and the strength of the evidence was shown by the thickness of the lines proportional. Conversely, intervention methods that are not connected by lines cannot be directly compared. The thinner lines indicate weaker connections, potentially reflecting marginal effects that lack statistical significance. Notably, the comparisons CRT vs. Sham and LF-rTMS vs. HF-rTMS have confidence intervals that include zero, indicating these effects are not statistically significant at the 5% level (Supplementary material: Appendix 2).

#### Inconsistency test

Inconsistencies across outcome measures were assessed using loop inconsistency tests, inconsistency models, and node-splitting methods. The results of the loop inconsistency tests indicated that the inconsistency of all triangular loops for HAMD was not significant (*p* > 0.05). The node-splitting method revealed that all outcome measures have no local inconsistency due to the direct comparison evidence and indirect comparison evidence were consistent (*p* > 0.05), suggesting a high level of reliability in the results.

#### The effects of different rTMS interventions on the PD-D

**Figure 4:**
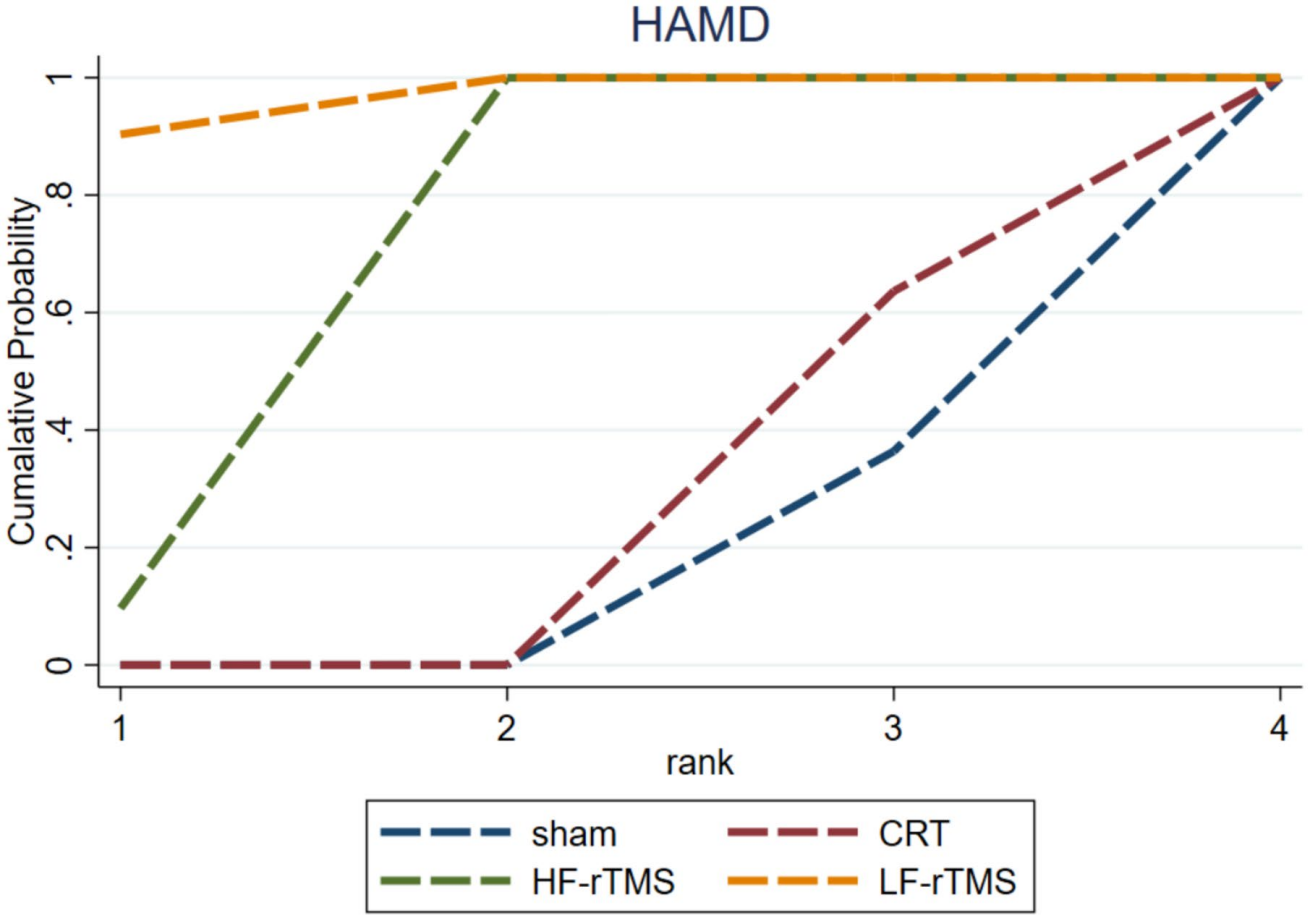
Surface under the cumulative ranking curve of different interventions.

As shown in Figure 4, the surface under the cumulative ranking curve (SUCRA) was applied to quantify the probability that each intervention ranks as the most effective. According to SUCRA, the probability ranking for improvement in HAMD was: LF -rTMS (96.7%), HF-rTMS (70.0%), CRT (21.2%), and sham (12.1%).

#### Adverse reaction

12 studies reported that some patients experienced transient mild symptoms such as headaches, neck pain, insomnia, and tinnitus during treatment. However, these symptoms were tolerable and showed significant improvement after adjusting the stimulation intensity or taking rest. Other studies did not report any additional adverse reactions.

### Outcomes of the subgroup analysis

#### subgroup analysis of different versions of HAMD scale

25 studies, involving 1,569 participants, evaluated the effectiveness of rTMS intervention in PD-D, using various versions of the HAMD scale. The treatment group demonstrated significantly greater efficacy than control group [MD = −3.92, 95% CI (−4.72, −3.58), *p* < 0.00001], and there is no significant heterogeneity was found (Chi^2^ = 31.02, *p* = 0.12; *I*^2^ = 26%; Supplementary material: Appendix 3). Considering the variability in HAMD scale usage across different studies, we analyzed this factor and conducted a subgroup analysis. Consistent with our hypothesis, the efficacy evaluation of rTMS yielded slightly varied results when different HAMD scales were used, and the HAMD-17 scale results most closely aligning with the final effect size.

#### Subgroup analysis of study region

A total of 26 studies from China, involving 1,461 participants, indicated that the treatment group was significantly superior to the control group [MD = −3.94, 95% CI (−4.28, −3.59), *p* < 0.00001], with no significant heterogeneity observed (Chi^2^ = 32.41, *p* = 0.05; *I*^2^ = 35%). The remaining 5 studies were conducted in other countries, involving 128 participants. These studies also demonstrated a significant difference between the treatment and control groups [MD = −2.33, 95% CI (−4.54, −0.12), *p* = 0.04], and no heterogeneity was detected (Chi^2^ = 0.57, *p* = 0.75; *I*^2^ = 0%%; Supplementary material: Appendix 3). Subgroup analysis indicated that the effect size of rTMS might be influenced by regional factors. Nevertheless, the overall findings demonstrated that rTMS was effective in significantly improving symptoms in patients with PD-D.

#### Subgroup analysis of second outcomes

##### BDI

6 studies, involving 203 participants, utilized the BDI scale as a measure for assessing the therapeutic efficacy of rTMS which was used as an intervention for PD-D. The overall analysis revealed no statistically significant difference in treatment effect between the rTMS and control groups (Chi^2^ = 6.82, *p* = 0.23; *I*^2^ = 27%), indicating low heterogeneity across the studies (Supplementary material: Appendix 3). Subgroup analysis revealed that the majority of studies utilized HF-rTMS as the intervention method, demonstrated a significant treatment effect [MD = −4.14, 95% CI (−5.94, −2.35), *p* < 0.00001].

##### UPDRS-I

As a commonly used scale for clinically assessing the efficacy of interventions, the UPDRS-I is often utilized to evaluate the severity of mental activities, behavioral, and mood disturbances in PD patients (29). In this review, we also utilized it to evaluate the efficacy of rTMS treatment. A meta-analysis of 7 studies, including 297 participants, demonstrated that rTMS exhibited a curative effect in improving UPDRS-I scores with statistical significance [MD = −0.82, 95% CI (−1.02, −0.63), *p* < 0.00001] (Supplementary material: Appendix 3). Subgroup analysis showed that the majority of the studies applied HF-rTMS, demonstrating a statistically significant treatment effect [MD = −0.84, 95% CI (−1.04, −0.64), *p* < 0.00001]. Conversely, two studies implemented LF-rTMS, which resulted in a non-significant effect [MD = −0.58, 95% CI (−1.32, −0.15,), *p* = 0.12]. The test for subgroup differences showed no statistical significance (Chi^2^ = 0.44, *p* = 0.51; *I*^2^ = 0%), suggesting that the efficacy difference between HF-rTMS and LF-rTMS was not pronounced. Nevertheless, the small number of LF-rTMS studies limits the ability to draw a conclusive comparison between the two protocols.

#### Subgroup analysis of other influencing factors

To account for factors that may influence assessment outcomes and potentially contribute to variability in results, we conducted a subgroup analysis using rTMS intervention parameters, such as targeting location, intensity, and total number of pulses, as potential influencing factors to explore their effects on the primary outcomes. Given that the included studies utilized different versions of the HAMD, which could introduce heterogeneity in the measurement of depression severity, we employed the Standardized Mean Difference (SMD) as the effect size metric for this subgroup analysis (Supplementary material: Appendix 3).

##### Targeting location

Although the subgroup analysis of targeting locations showed that rTMS appears to be more effective in alleviating depressive symptoms when applied to the RDLPFC [SMD = −0.68, 95% CI (−0.90, −0.45), *p* < 0.00001], the differences among subgroups were not statistically significant (Chi^2^ = 5.02, *p* = 0.28; *I*^2^ = 20.4%). The funnel plot appeared approximately symmetrical, suggesting that the overall findings were minimally influenced by publication bias. However, the limited number of studies specifically targeting the double dorsolateral prefrontal cortex (DDPLFC) may reduce the stability of the conclusion and highlights the need for additional research to validate the observed effects.

##### Intensity

Subgroup analysis of rTMS intensity indicated that the 80–90% resting motor threshold (RMT) produced the largest effect size for reducing depressive symptoms [SMD = −0.74, 95% CI (−0.92, −0.56), *p* < 0.00001], but the differences across subgroups were not statistically significant (Chi^2^ = 2.79, *p* = 0.43; *I*^2^ = 0%). Furthermore, the funnel plot appeared approximately symmetrical, indicating minimal influence from publication bias. Since the included studies focused on an intensity range of 80–110%, with a limited number of studies investigating higher intensity, indicating that additional research is needed to enhance the reliability of the findings.

##### Total pulses

Interestingly, the subgroup analysis demonstrated that rTMS was significantly more effective in alleviating depressive symptoms when the total number of pulses was less than or equal to 1,200 [SMD = −0.91, 95% CI (−1.13, −0.68), *p* < 0.00001], compared to pulse numbers exceeding 1,200. Moreover, the difference between two groups reached statistical significance (Chi^2^ = 4.29, *p* = 0.04; *I*^2^ = 76.7%), suggesting that a lower cumulative pulse count may potentiate therapeutic efficacy.

## Discussion

This systematic review and network meta-analysis of 35 RCTs (2,353 participants) demonstrates that both HF-rTMS and LF-rTMS are effective in alleviating depressive symptoms in patients with PD. LF-rTMS appears to offer greater benefits, as evidenced by a higher SUCRA probability (96.7% vs. 70.0% for HF-rTMS). These findings suggest that LF-rTMS, which inhibits cortical excitability, may be particularly effective in downregulating hyperactive neural circuits associated with PD-D. Clinically, LF-rTMS could serve as a valuable adjunctive therapy, especially for patients with poor response to pharmacological treatments.

In this study, we observed variations in the scales used to assess depression during rTMS treatment across the included studies. These scales included the HAMD-17, HAMD-24, and the Hamilton Rating Scale for Depression (HRSD). Notably, various versions of the HAMD scale may have a certain impact on the final result. To address this, we conducted further analysis, which revealed that scores from the HAMD-17 scale aligned more closely with the overall findings. This may be due to several factors: As the most commonly used scale to evaluate the efficacy of antidepressants (30, 31), HAMD-17 is widely recognized for its reliability and comprehensive assessment, so the number of studies utilizing this scale has increased. Additionally, some studies may exclude patients with severe depression, which would make a more concentrated range of depressive severity among participants (32), and the therapeutic effect after receiving the intervention may be more similar. A meta-analysis (33) has suggested that the HAMD-6 may exhibit superior clinical properties compared to the HAMD-17, with its efficacy well-supported in clinical settings. This makes it a promising instrument for assessing core depressive symptoms in hospitalized patients and potentially assisting in treatment decisions (34–36). Employing multiple versions of depression assessment scales could improve the objectivity and robustness of efficacy evaluations. Among these tools, the BDI, well known for its high sensitivity and specificity, serves as a valuable tool for early detection of depression in PD patients (37, 38). In our analysis, we also included studies that used the BDI in selecting outcome measures to reduce the potential for measurement error. Nevertheless, the observed differences in efficacy may be constrained by the limited number of included studies, highlighting the need for further high-quality research to enhance the precision and generalizability of rTMS outcome assessments. It is also important to note that the use of different scales can introduce measurement bias across populations from diverse regions and cultural backgrounds. Since the majority of the included studies were conducted in China, our subgroup analysis indicated that while both studies from China and those from other countries demonstrated significant intervention effects, the effect size was larger in Chinese studies. This regional difference, potentially attributable to cultural or methodological factors, may limit the generalizability of our findings. Therefore, such variability should not be overlooked (39).

Recent studies have demonstrated that rTMS enhances global brain functional network connectivity, particularly by strengthening the connections between the prefrontal cortex and limbic structures such as the amygdala and cingulate cortex, thereby facilitating top-down modulation of emotional responses (40–43). Furthermore, rTMS promotes the release of key neurotransmitters, including dopamine and serotonin, which play a critical role in the amelioration of depressive symptoms (44–46). In addition, rTMS increase synaptic plasticity by inducing synaptogenesis and improving neurocircuitry involved in emotional regulation (47, 48). Based on the above, we analyzed various rTMS parameter settings, including the stimulation target, frequency, intensity, and total number of pulses. The dorsolateral prefrontal cortex (DLPFC) plays a central role in regulating emotional, social, and cognitive functions (49). Studies have shown that emotional regulation in individuals with depression often shifts from the lateral frontal pole cortex to the DLPFC (50, 51). Moreover, individuals with major depressive disorder exhibit reduced frontoparietal network connectivity, with the DLPFC implicated in this dysfunction. Depression is generally characterized as an exaggerated response to external stress and exhibits specific symptoms. It has been associated with hyperactivity of the RDLPFC (52–55). Several studies (26, 56, 57) have indicated that low-frequency stimulation targeting the RDLPFC, the most commonly used region in antidepressant rTMS protocols, has shown favorable efficacy, the findings are similar to our analysis results. Considering the limited understanding of the specific neural circuitry involved in PD with depression, as well as the mechanisms through which rTMS exerts its neuromodulatory effects, it is hypothesized that cortical activation may extend beyond the stimulated regions and propagate through neural networks to distant areas (58). LF-rTMS targeting the hyperactive RDLPFC can induce effects resembling long-term depression (LTD), leading to the suppression of cortical excitability. This inhibitory effect may contribute to the downregulation of hyperactive neural circuits associated with depressive symptoms (59). LF-rTMS may promote striatal dopamine release via the fronto-parietal-striatal-cortical pathway, thereby improving the neurochemical basis of PD-D (26).

The parameters of rTMS, particularly stimulation intensity and total pulse number, were also preliminarily analyzed in this study. Rossini et al. (60) compared stimulation intensity at 80 and 100% of the RMT, finding that 100% RMT produced better antidepressant effects. Since stimulation intensity is adjusted relied on the minimum threshold needed to induce a minimal muscle contraction response, it varies among individuals. Caulfield et al. (61) developed a new approach, termed “A Personalized E-field X Motor Threshold” (APEX MT), to make stimulation intensity more personalized and accurate. In addition, the findings indicated that rTMS yielded optimal antidepressant effects with 1,200–1,500 high-frequency pulses or 360–450 low-frequency pulses, with no additional benefits observed from increasing the total pulse count (62, 63). This phenomenon may stem from a plateau effect in pulse quantity, whereby exceeding a certain threshold does not further enhance cortical excitability and may even induce inhibitory effects or neural fatigue (64, 65). Generally, the optimal number of pulses may be influenced by the specific rTMS frequency utilized, as well as individual patient response and tolerance. Achieving the appropriate balance is crucial for maximizing therapeutic efficacy while minimizing patient burden. The distribution and intensity of the electric field generated within the target brain region during TMS are affected by coil positioning, as the coil induces intracortical currents that modulate neuronal firing patterns (66). Currently, anatomical localization methods rely on scalp landmarks, but these are susceptible to individual anatomical variability. Neuronavigated localization, utilizing neuronavigation systems, enables more precise coil placement; however, existing studies have not conclusively demonstrated the significant superiority of neuronavigation (67). Notably, Moser et al. used a novel multi-task deep neural network to rapidly determine the optimal coil position for inducing maximal electric field at the target site (68).

Compared to existing meta-analyses, our study not only provides a more detailed analysis of rTMS parameters (frequency, target location, stimulation intensity, and total pulses count), but retrospectively assesses the impact of rating scales on outcomes, thereby offering clinical guidance for rTMS use in PD-D and insights into assessment scale selection in practice.

## Limitations

This study has several limitations: Influenced by the included studies, the range of TMS modalities investigated is limited, and the predominance of studies from Chinese databases, combined with cultural differences and varied evaluation scales may introduce bias, potentially affecting the robustness of the results. Additionally, both the intervention and control groups received antidepressant medications in most included studies, which may affect the accurate evaluation of the efficacy of TMS. Furthermore, the heterogeneity in rTMS parameter combinations and the lack of information on coil positioning and localization methods restrict the standardization of treatment recommendations. The absence of reported Hoehn–Yahr stages in baseline characteristics in most studies further hinders the evaluation of disease severity’s impact on treatment outcomes. These limitations underscore the need for more high-quality, multi-national randomized controlled trials in future research, in order to enhance the generalizability, reliability, and clinical applicability of the findings.

## Conclusion

This study indicates that both HF-rTMS and LF-rTMS are effective for improving depressive symptoms in PD patients, with LF-rTMS potentially exhibiting greater benefits compared to other interventions. Moreover, the application of specific intervention parameters, such as targeting applying no more than 1,200 pulses per session, may yield enhanced outcomes. Nevertheless, further high-quality RCTs are required to validate and refine this conclusion.

## Data Availability

The data that support the findings of this study are available from the corresponding author upon reasonable request.
