# Supplementary material for: Comparative efficacy of different modalities of transcranial magnetic stimulation for treating Parkinson’s disease with depression: a systematic review and network meta-analysis
Source: Front Neurol. 2025 Jul 31;16:1627932. doi: 10.3389/fneur.2025.1627932 (PMC12352168; doi:10.3389/fneur.2025.1627932)

**Appendix**

**Catalogues**

[**Appendix 1: Search strategy** 1](#_Toc203084445)

[**Appendix 2: Forest Plot Comparing different rTMS interventions** 4](#_Toc203084446)

[**Appendix 3: Subgroup analysis** 5](#_Toc203084447)

[**3.1 Different versions of HAMD scale** 5](#_Toc203084448)

[**3.2 Different study regions** 7](#_Toc203084449)

[**3.3 BDI** 8](#_Toc203084450)

[**3.4 UPDRS-Ⅰ** 9](#_Toc203084451)

[**3.5 The different parameters setting of rTMS on primary Outcomes** 10](#_Toc203084452)

# **Appendix 1: Search strategy**

**CBM:**

#1 “帕金森病”[不加权:扩展]

#2 “经颅磁刺激” [不加权:扩展]

#3 “帕金森”[常用字段:智能] OR 震颤麻痹[常用字段:智能]

#4 “TMS”[常用字段:智能]

#5 “随机”[摘要:智能] OR “临床观察”[摘要:智能] OR “疗效观察”[摘要:智能] OR “临床研究”[摘要:智能]

#6 ((#1) OR (#3))

#7 ((#2) OR (#4))

#8 ((((#6) AND (#7))) AND (#6))

**CNKI:**

（主题：帕金森 + 帕金森病 + 震颤麻痹）AND（主题：经颅磁刺激 + TMS）AND（摘要：随机 + 临床观察 + 疗效观察 + 临床研究(精确)）

**Cochrane:**

#1 MeSH descriptor: [Parkinson Disease] explode all trees

#2 (paralysis agitans):ti,ab,kw OR (parkinson s disease):ti,ab,kw OR (shaking palsy):ti,ab,kw OR (Parkinsonism):ti,ab,kw

#3 MeSH descriptor: [Transcranial Magnetic Stimulation] explode all trees

#4 (magnetic stimulation transcranial):ti,ab,kw OR (stimulation transcranial magnetic):ti,ab,kw OR (transcranial magnetic stimulation repetitive):ti,ab,kw OR (TMS):ti,ab,kw

#5 #1 OR #2

#6 #3 OR #4

#7 #5 AND #6

**Embase:**

#1 'parkinson disease'/exp

#2 'paralysis agitans':ab,ti OR 'parkinson s disease':ab,ti OR 'shaking palsy':ab,ti OR 'parkinsonism':ab,ti

#3 'transcranial magnetic stimulation'/exp

#4 'magnetic stimulation transcranial':ab,ti OR 'stimulation transcranial magnetic':ab,ti OR 'transcranial magnetic stimulation repetitive':ab,ti OR 'tms':ab,ti

#5 'randomized controlled trial':ab,ti OR 'randomized':ab,ti OR 'placebo':ab,ti

#6 #1 OR #2

#7 #3 OR #4

#8 #5 AND #6 AND #7

**Pubmed:**

#1 "Parkinson Disease"[Mesh]

#2 (((Paralysis Agitans[Title/Abstract]) OR (Parkinson's Disease[Title/Abstract])) OR (shaking palsy[Title/Abstract])) OR (Parkinsonism[Title/Abstract])

#3 "Transcranial Magnetic Stimulation"[Mesh]

#4 (((Magnetic Stimulation, Transcranial[Title/Abstract]) OR (Stimulation, Transcranial Magnetic[Title/Abstract])) OR (Transcranial Magnetic Stimulation, Repetitive[Title/Abstract])) OR (TMS[Title/Abstract])

#5 randomized controlled trial[Publication Type] OR randomized[Title/Abstract] OR placebo[Title/Abstract]

#6 #1 OR #2

#7 #3 OR #4

#8 #5 AND #6 AND #7

**VIP:**

((((((((题名或关键词=帕金森 OR 题名或关键词=帕金森病) OR 题名或关键词=震颤麻痹) AND (题名或关键词=经颅磁刺激 OR 题名或关键词=TMS)))) AND (((摘要=随机 OR 摘要=临床观察) OR 摘要=疗效观察) OR 摘要=临床研究))))

**WanFang:**

检索表达式：主题:(帕金森 OR 帕金森病 OR 震颤麻痹) and 主题:(经颅磁刺激 OR TMS) and 摘要:(随机 OR 临床观察 OR 疗效观察 OR 临床研究)

**Web of Science:**

#1 TS=(Parkinson Disease OR paralysis agitans OR Parkinson’s disease OR shaking palsy OR Parkinsonism)

#2 TS= (Transcranial Magnetic Stimulation OR "Magnetic Stimulation*, Transcranial" OR "Stimulation, Transcranial Magnetic")

#3 AB= (randomize OR placebo OR RCT)

#4 #1 And #2 And #3

# **Appendix 2: Forest Plot Comparing different rTMS interventions**


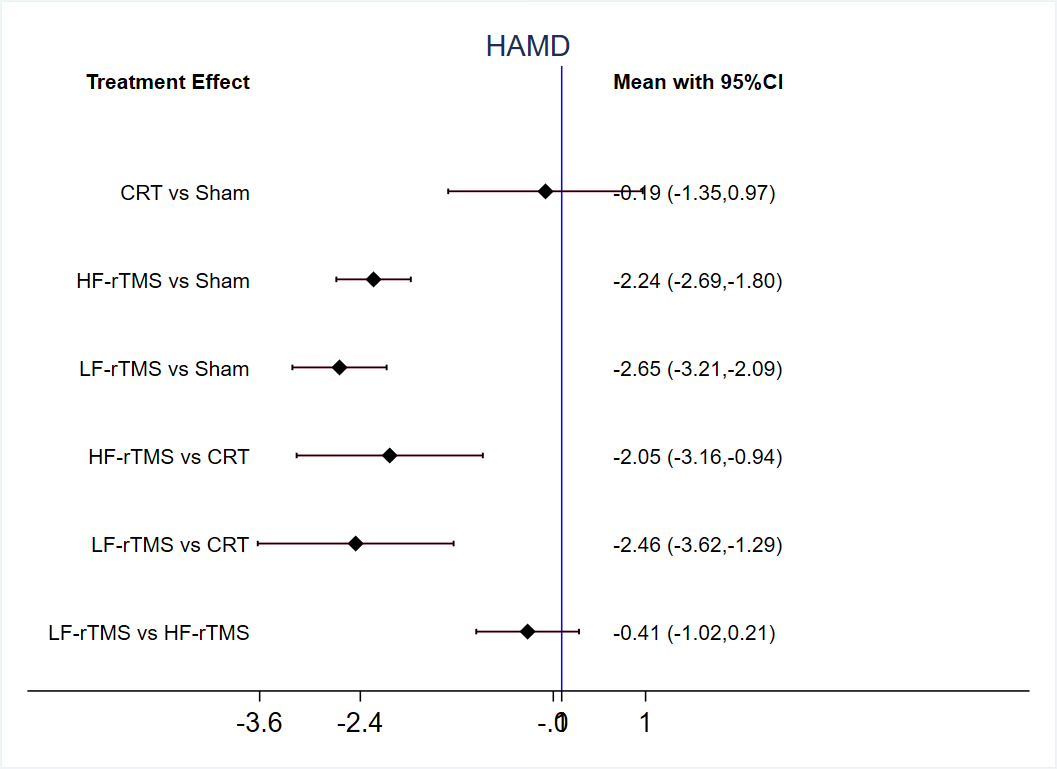


# **Appendix 3: Subgroup analysis**

## **3.1 Different versions of HAMD scale**

**Forest plot**

**
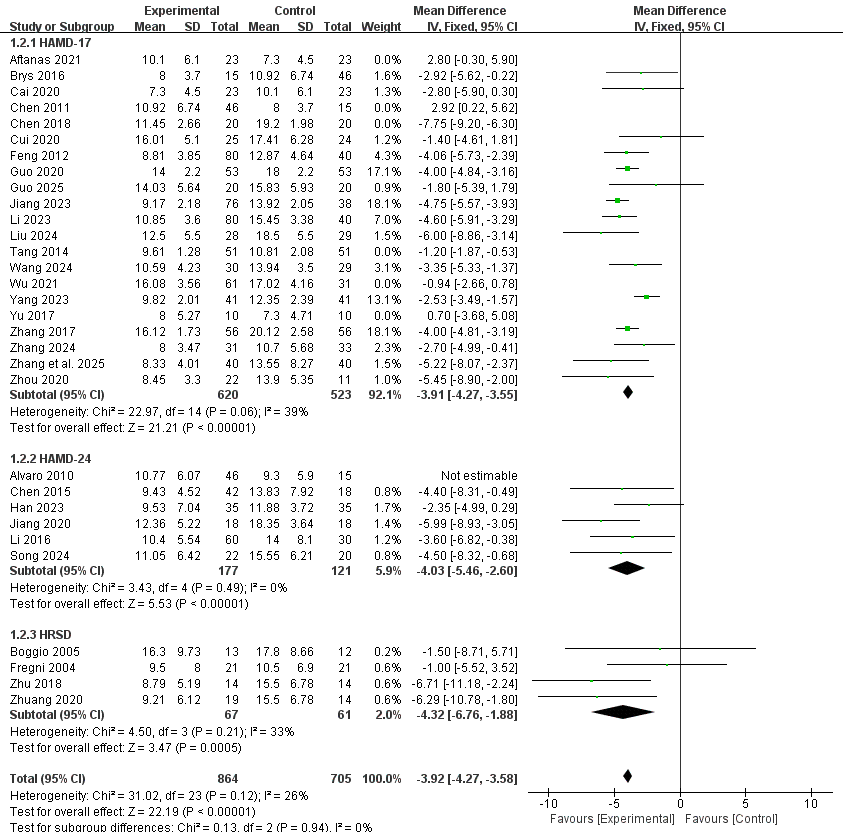
**

**Funnel plot**

**
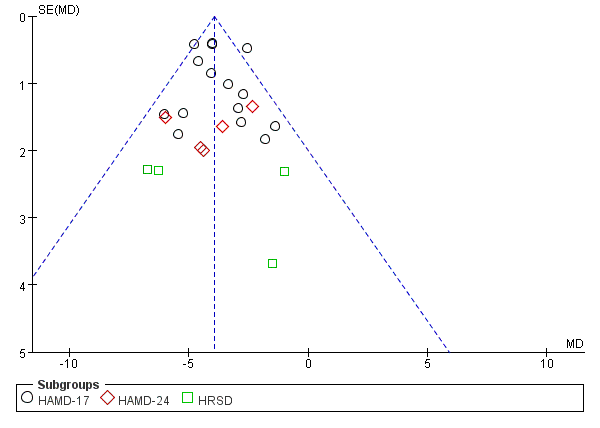
**

## **3.2 Different study regions**

**Forest plot**

**
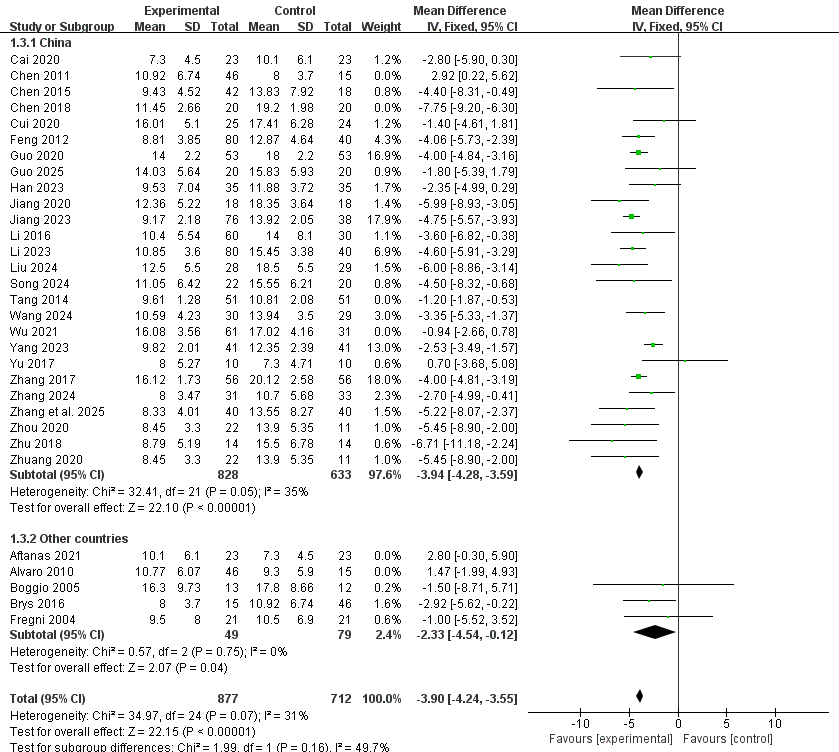
**

**Funnel plot**

**
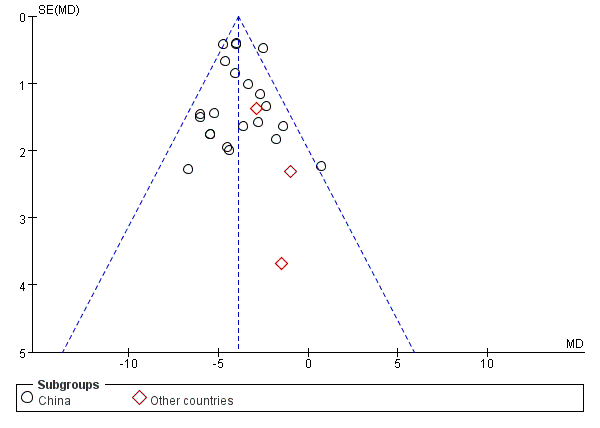
**

## **3.3 BDI**

**Forest plot**

**
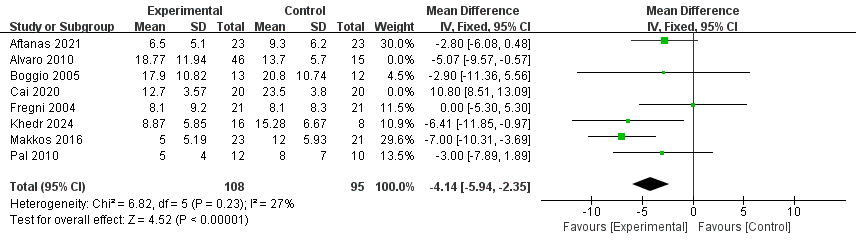
**

**Funnel plot**

**
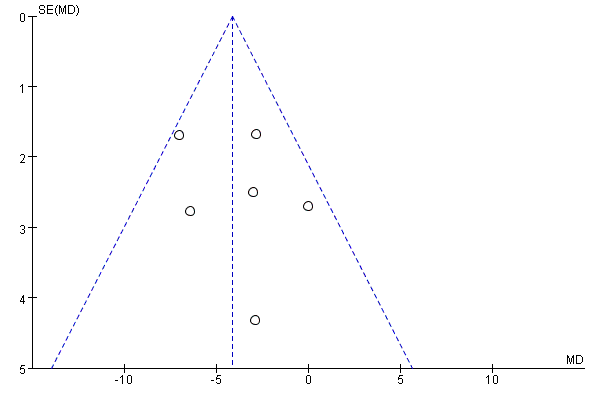
**

## **3.4 UPDRS-Ⅰ**

**Forest plot**

**
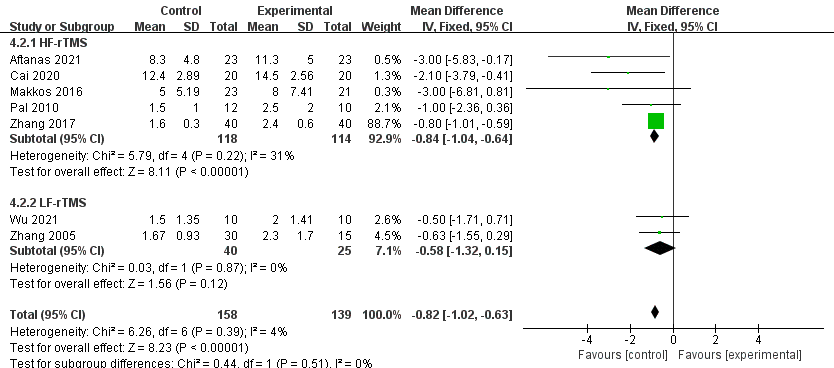
**

**Funnel plot**

**
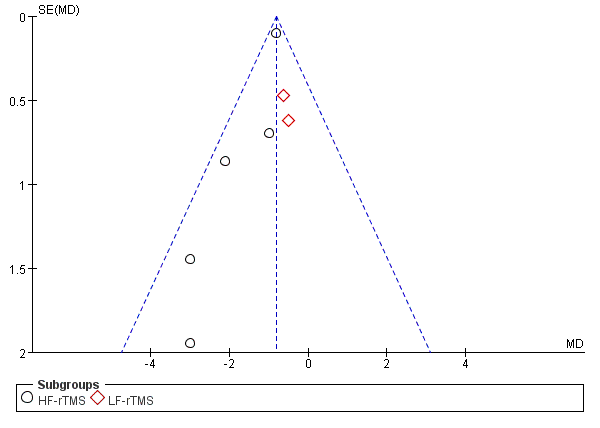
**

## **3.5 The different parameters setting of rTMS on primary Outcomes**

**3.5.1 Location**

**Forest plot**


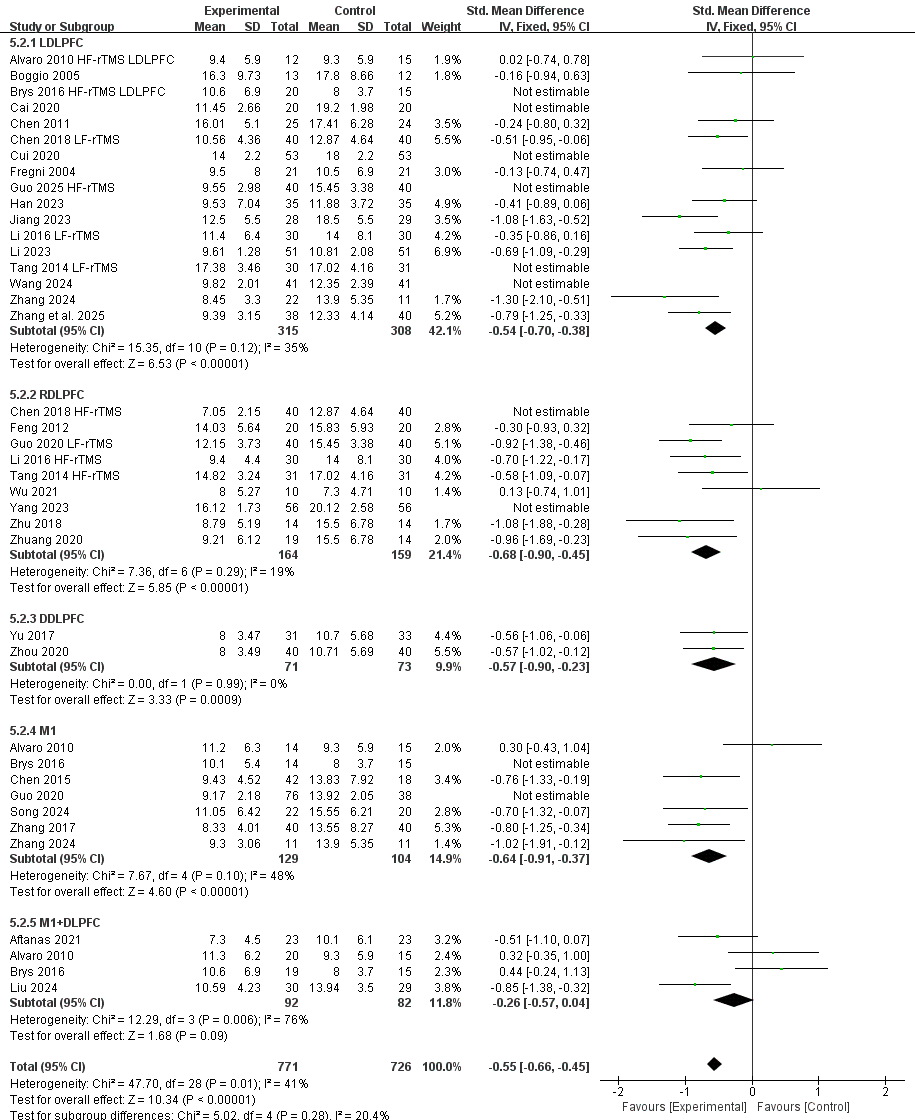


**Funnel plot**


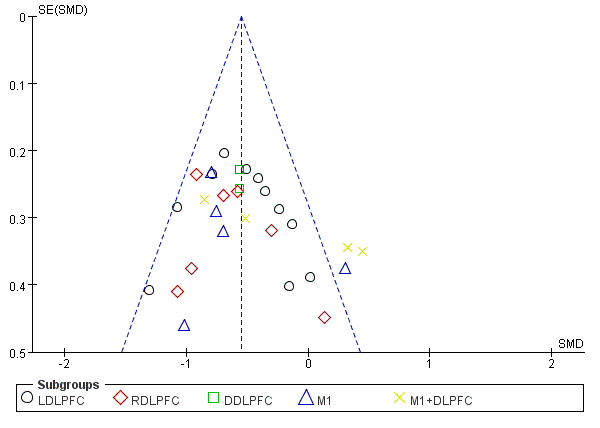


**3.5.2 Intensity**

**Forest plot**


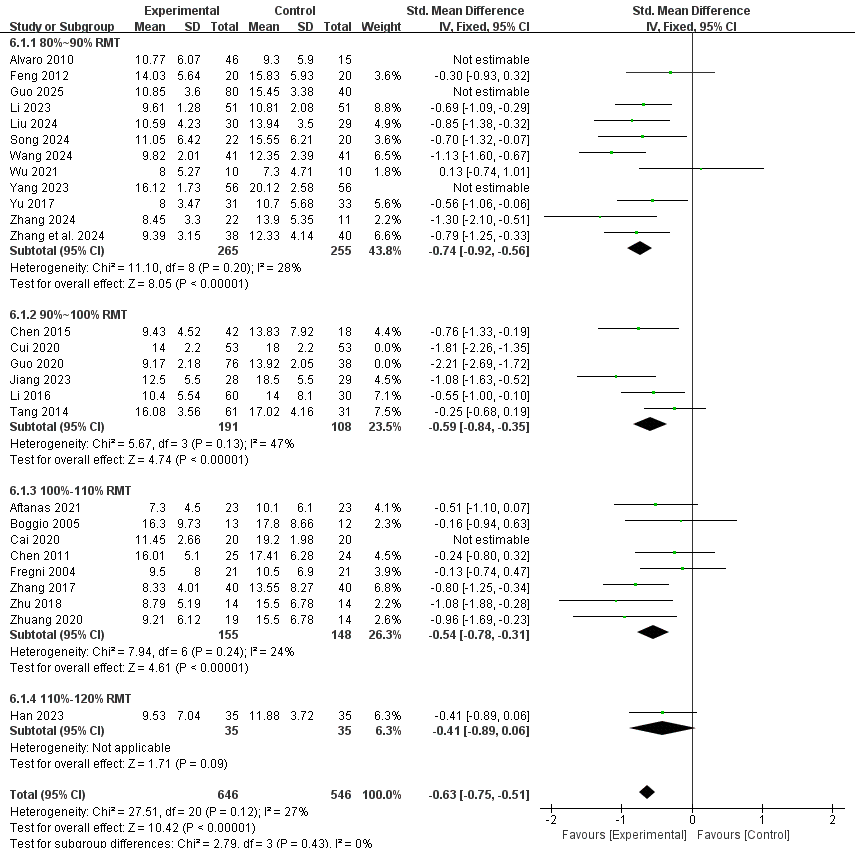


**Funnel plot**


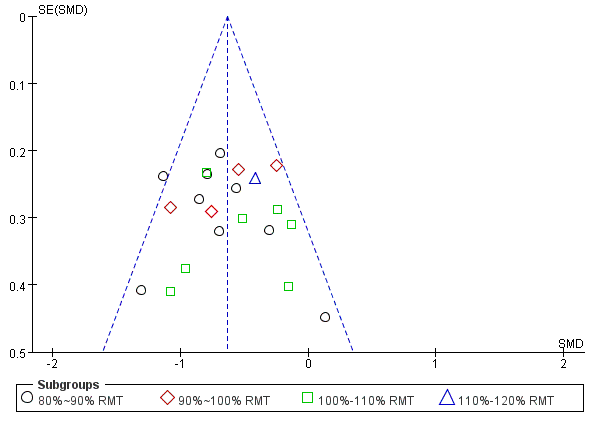


**3.5.3 Total pulses**

**Forest plot**


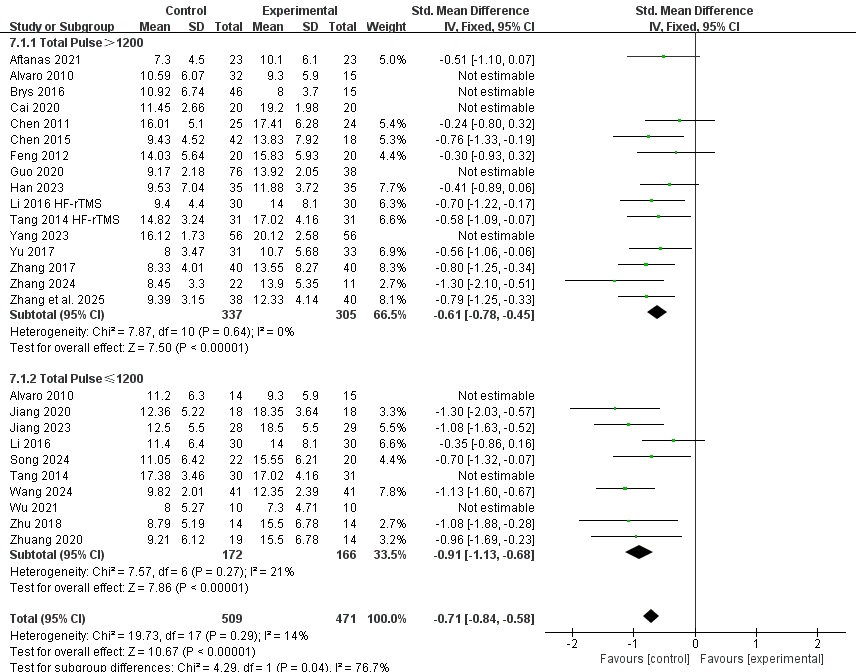


**Funnel plot**


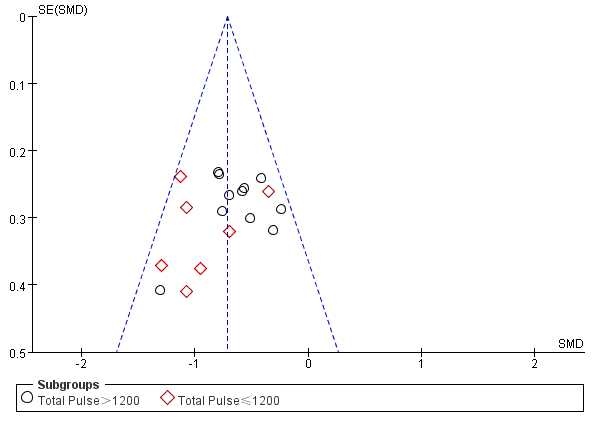

Supplement: Supplementary file 1 [file Supplementary_file_1.docx]
